# Differences in Student Brain Activation from Digital Learning Based on Risk of Digital Media Addiction

**DOI:** 10.3390/ijerph182111061

**Published:** 2021-10-21

**Authors:** GyeongAe Seomun, Wonjung Noh

**Affiliations:** 1 College of Nursing, Korea University, Seoul 02841, Korea; seomun@korea.ac.kr; 2 BK21FOUR R&E Center for Learning Health Systems, Korea University, Seoul 02841, Korea; 3 College of Nursing, Gachon University, Incheon 21936, Korea

**Keywords:** addictive behavior, attention, brain, relaxation, students

## Abstract

Although digital media usage is prevalent among middle school students, the safety of digital media-based learning activities for students at risk of digital media addiction is unknown. The goal of this study was to evaluate the differences in students’ brain activity in relation to their risk of digital media addiction. The study was quasi-experimental, with a pre- to post-test control group design. The study participants included 83 middle school students who were engaged in digital learning. We measured their brainwaves to evaluate brain activity using a PolyG-I (LAXTHA Inc.). We found no statistically significant differences in the location of the attention index between the two groups before and after digital learning. However, there were statistically significant differences between the two groups in the P3, P4, and F4 locations of the relaxation index. These results indicate that students at risk of digital media addiction may experience learning difficulties. These results can be used to guide healthcare professionals in developing digital learning programs that are safe for students and to also verify the effects of these programs.

## 1. Introduction

New technologies have reshaped learning and education. Digital learning is widespread, facilitated by devices such as personal computers, laptops, and smartphones in school and at home [1]. Although the benefits of digital learning are appealing in terms of achieving learning goals, the guardians and teachers of students participating in digital learning are concerned about the potential side effects on these adolescents’ mental health because of the increase in device usage time [2,3]. In particular, there is increasing concern that the increase in device usage time will lead to digital dependence or addiction. Concerns related to the potential for health problems related to digital learning include physical health problems, such as musculoskeletal disorders and headache, and psychological health problems, such as depression, stress, and concentration disorders, which may emerge from early childhood onwards [4,5,6,7,8,9]. On the other hand, research on the effect of increasing digital device usage time on the health of adolescents is insufficient.

Previous studies have reported that the use of digital devices can cause not only physical but also psychological symptoms. Student health problems related to the use of digital devices include physical symptoms such as eye health and musculoskeletal health [10,11]. Other studies have shown that students at risk of digital media addiction are most aware of their psychological symptoms vs. other types of symptoms [12]. Digital media addiction was recently bringing about symptoms in children and adolescents; it was excessive usage of digital devices such as tablets, smartphones, etc. [13]. Among the types of digital media addiction, cell phone addiction is related to adolescents’ mental health and physical health [14]. The act of using a digital device, watching television for example, has effects similar to those of substance abuse and increases children’s and adolescents’ passivity about receiving the attention they need [15].

In addition, previous studies have shown that the increasing use of digital devices has raised concerns regarding physical and psychological health problems, and even quality of life [16]. In addition, this increase may lead to digital media addiction. For instance, people who use the internet more frequently have an increased risk of poor physical health [17]. Smartphone use can also have harmful effects on both the interpersonal relationships and the psychological health of adolescents [18]. Because the type of digital device used and the time spent on the device can affect adolescents’ health, their risk of digital media addiction and the potential implications for their health must be considered. Students may experience attention and relaxation difficulties when they use digital devices, which can be assessed by measuring the levels of brain activity when learning how to use digital devices. There are different opinions regarding digital learning and its effects; thus, it is necessary to measure brain activity using electroencephalography (EEG) while students are learning how to use digital devices to help clarify these controversies.

EEG is a useful tool for measuring learning effects while students are learning in real time [19,20], as well as for measuring their levels of attention and relaxation [21]. EEG is a non-invasive, inexpensive, safe, and easily operated method frequently used to examine learning effects through brain activity in children and adolescents [22,23]. Previous studies have used EEG to measure attention and relaxation in children and adolescents with attention deficit hyperactivity disorder (ADHD) [24]. For assessing attention, a previous study measured the ratio of the sensory motor rhythm (SMR) ~mid-β waves to theta waves (RSMT) [25,26]. In addition, relaxation was measured using the ratio of α to high-β waves (RAHB) [26,27]. EEG is a suitable tool for identifying attentive and relaxed states in adolescent students’ brains during the learning process.

Herein, we compared the brain activity and the levels of attention and relaxation while using a digital device for learning between students at different risk levels for digital media addiction. The risk levels for digital media addiction were assessed in the survey, and then, data on students’ brainwaves were measured by EEG.

### Purpose

The aim of this study was to compare the brain activity and the levels of attention and relaxation while using a digital device for learning between students at risk of digital media addiction (risk group (RG)) and students not at risk (without-risk group (WRG)).

## 2. Materials and Methods

### 2.1. Design

This study was a case–control study comparing brain activity and levels of attention and relaxation between RG and WRG.

### 2.2. Participants

A convenience sampling approach was used to generate the study population. A total of 83 middle school students (54 students with no risk and 29 students at risk of digital media addiction) in Korea were selected for the study. We first targeted the middle schools and then obtained the principals’ consent for the recruitment of students for this study. The inclusion criteria for participants were: (1) students with prior learning experience using a digital device such as a computer, smartphone, or television for more than one year; and (2) students who qualified for an EEG due to the absence of a cerebral disease and who did not drink coffee or soda and had not used any haircare substances within two hours prior to the procedure.

The sample size was calculated using G Power 3.1.3, which was also used to determine that this study required a Wilcoxon–Mann–Whitney test (two groups) analysis with an effect size of 0.62, a power of 0.8, and a significance level of 0.05, which resulted in the requirement for a total sample size of at least 78 students (group 1 = 26, group 2 = 52). The effect size of 0.62 was chosen based on a previous research study on the baseline of neurofeedback among ADHD children [28]. In the current study, the sample size requirement was met as 83 students were enrolled. When the effect size was calculated with the number of subjects that actually participated in the study, the number of subjects was considered appropriate because the calculated effect size of 0.61 was similar to that assumed before the study was conducted.

### 2.3. Procedure

Data were collected using two methods: survey and brainwave measurement. First, data on the student characteristics and levels of digital media addiction were collected via self-report. Students were asked to complete a questionnaire on their experiences with digital learning, the time they had previously spent participating in digital learning, and their health status, and were also asked to complete the Diagnosing Smartphone Addiction [29] and Diagnosing Online Game Addiction survey [30].

After the self-report survey, data on students’ brainwaves were measured using the PolyG-I computerized EEG meter (LAXTHA Inc., Daejeon, Korea). Researchers trained in EEG examination performed the measurements in experimental rooms in the schools between 15 November and 7 December 2014. During the brainwave measurements, the students sat in a room with minimal noise with the electrodes attached to their scalps. They maintained a stable state with their eyes closed during the measurements. The electrodes were attached to eight sites on the scalp as per a previous study and in accordance with the International 10/20 System of Electrode Placement [31]. The measurements consisted of five steps. In the first step, brainwaves of students in the stable state were measured for 6 min. During this step, the students maintained a stable state without moving their bodies with their eyes open. This was followed by a 1 min rest period, constituting the second step; when continuous measurements were made without a rest time, it was difficult to separate the exact interval for stimulation and response, which may create difficulties in data analysis, so the 1 min rest period was used to avoid this scenario [32]. In the third step, the students had 6 min of learning time using a digital media device, followed by a 1 min rest period as the fourth step. During the learning time, the students studied using digital textbooks, which included audiovisual images on a digital media device such as a laptop computer. The content comprised social science subjects. The students solved pop-up quizzes, but these results were not used for analysis in this study. In order to focus on the effect of the learning activity itself on brainwaves, we tried to minimize the stress and learning gap caused by pop-up quizzes for students. In the final step, the students’ brainwaves were measured in the stable state for 6 min, the same as the first step. For brainwave data analysis, we used 4 min of brainwaves out of the 6 min measured before learning. The 1 min after the measurement started and 1 min before the end of the measurement were excluded from analysis. In addition, 4 min of brainwaves was used out of the 6 min of brainwaves measured after learning. As with before learning, the 1 min after the measurement started and the 1 min before the end of the measurement were excluded from the analysis. In this way, we were able to obtain reliable data by preventing data contamination due to the inflow of artifact waves that may occur during the movement from one step to another.

### 2.4. Measures

#### 2.4.1. General Characteristics

The participants completed a questionnaire about their characteristics, including information about their sex (51.8% male, 48.1% female), age (mean 14.69 y), digital learning status, and health status. To assess digital learning status, information regarding past experiences with digital learning, time spent participating in digital learning, and the types of digital devices used was collected. To assess health status, the students’ health status and feelings of fatigue or pain when using digital devices were investigated.

#### 2.4.2. Allocation of Participants into the RG and WRG

The definition of digital media addiction included the overuse of smartphones and digital devices to watch television, search the internet, or play online games [33]. We defined the risk of digital media addiction as belonging to one of the potential risk groups for smartphone or online game addiction according to previous research [12]. The students were assigned to the WRG if they were not included in either the smartphone or the online game addiction groups. Students were assigned to RG if either their score for Diagnosing Smartphone Addiction (DSA) was higher than 46/60 points or their score on the Diagnosing Online Game Addiction Scale (DOGA) was higher than 46/80 points.

For the allocation of participants into the groups, the risk of digital media addiction was measured using the DSA and DOGA from the National Information Society Agency of Korea (NIA) [29,30]. These instruments were developed by the NIA to consistently measure digital media addiction through self-assessment and to provide appropriate interventions at the national level.

The DSA scale consists of 15 items, with a response scale ranging from 1 (strongly disagree) to 4 (strongly agree). The NIA guidelines define general users and potential at-risk users in smartphone addiction groups as those who obtain scores of less than 35 points and 36–45 points, respectively [29]. Cronbach’s α for this scale in this study was 0.86.

The DOGA scale consists of 20 items, with a response scale ranging from 1 (strongly disagree) to 4 (strongly agree). The NIA guidelines define general users and potential at-risk users in online game addiction groups as those who obtain scores of less than 35 points and 36–45 points, respectively [30]. Cronbach’s α for this scale in this study was 0.91.

#### 2.4.3. Effects of the Levels of Attention and Relaxation on Brainwaves

Brainwaves were measured and the level of attention was analyzed using the ratio of SMR ~mid-β waves to theta waves (RSMT) using the following formula: level of attention = (power ratio of SMR + M-β)/θ [25]. The sensory motor rhythm (SMR) and M-β wave values reflect attention: participants who had attention deficits were also shown to have a low level of attention. The level of relaxation was analyzed using the ratio of α to high-β waves (RAHB) using the following formula: level of relaxation = (power ratio of α)/(H-β) [25]. The H-β wave showed the participants’ stress level, while the α wave showed the participants’ relaxation level. These two indicators were relative values ranging from 0 to 1. A previous study used these indicators to determine the effects of e-learning in university students [20].

### 2.5. Data Analysis

Data analysis was conducted using STATA version 16.0 (STATA Corp LP, College Station, TX, USA) as follows: first, the characteristics of the two groups were analyzed using descriptive statistics; categorical variables were expressed as numbers and percentages and continuous variables as mean ± SD. The differences between groups were evaluated using χ^2^ tests, whereas Wilcoxon rank-sum test was used to compare the differences in brainwave activity between the two groups. Statistical significance was considered in the case of *p* < 0.05.

### 2.6. Ethical Consideration

Ethical approval was obtained from the University Institutional Review Board (Seoul, Korea). After receiving approval, we obtained the consent of the middle schools’ principals to recruit participants for this study. We then sent a letter requesting participant consent and an explanation of the study to the students’ guardians. We received consent forms signed by the students and their guardians before the study was conducted. The students voluntarily participated in this study and provided written informed consent. The consent form stated that the participants could withdraw from the study at any time and that their information would only be used for the present study.

## 3. Results

### 3.1. General Characteristics of the Study

The characteristics of the 83 students are listed in Table 1. Tests for homogeneity between the RG and WRG showed that the groups were homogenous for all variables. The WRG consisted of 54 students (28.9% male, 36.1% female), and the RG contained 29 students (22.9% male, 12.0% female). More than half of the students had no prior experience with digital learning (36.1% of the WRG, 19.3% of the RG). Over 50% of the students had experienced fatigue or pain during digital device use (34.9% of the WRG, 20.5% of the RG).

### 3.2. Brain Activation in Relation to Attention and Relaxation

Table 2 and Table 3 show the differences in the levels of attention and relaxation for each electrode position and group before and after digital learning. Table 2 shows the levels of attention of the two groups. Before digital learning, none of the electrode locations differed significantly between the two groups. Similar results were obtained after digital learning. The variances between the measures taken before and after digital learning did not differ significantly between the groups.

Table 3 shows the differences between before and after digital learning in the level of attention and relaxation between the two groups. Among the variances between before and after digital learning, a statistically significant difference at location F3 (left frontal lobe; t = −2.16, *p* = 0.031) and F4 (right frontal lobe; t = −2.39, *p* = 0.017) was observed in the relaxation index between WRG and RG.

## 4. Discussion

In the current study, no differences in the attention levels between RG and WRG were found. Although the RG showed lower levels of attention than the WRG, the difference was not statistically significant. However, the RG exhibited significantly different levels of relaxation in the parietal lobes before and after digital learning. In future research, it will be necessary to plan a research design that also considers time.

Attention is defined as the degree to which thoughts are gathered [34], which is essential for learning. In the present study, we found no statistically significant difference in attention between the two groups. However, previous studies have reported contradictory results. One study found that digital learning did not affect the attention of learners based on their brain activity [35], whereas another study reported digital learning to be more effective at encouraging students to concentrate while studying [36]. Due to the results that digital learning has a positive effect, such as increasing memory, and the results of conflicting studies on the negative effects, such as increased attention-deficit symptoms, the results of the effect of digital learning on brain health are controversial [37]. Since this is the same not only for adolescents but also for adults, further investigation is needed to identify the mechanism by which digital learning affects brain health and to determine the effect of digital learning on attention. In addition, since we compared WRG to RG, further research into the addiction group is necessary to measure the differences in attention levels.

The comparison of the measurements obtained before and after digital learning showed that the levels of relaxation at the F3 (left frontal lobe) and F4 (right frontal lobe) location differed significantly between the two groups. After digital learning, the WRG showed lower relaxation levels, whereas the RG showed higher relaxation levels. Relaxation is defined as an indication of a comfortable state [34]. For levels of relaxation to be elevated, either H-β waves are inactivated when emotionally unstable, or α waves are activated when the brain is resting [17]. This means that an excessive increase in the levels of relaxation can desensitize brain activity, so it can increase the risk of learning difficulty. As the frontal lobe is associated with intelligence, concentration, and short- and long-term memory [38], this result can be interpreted as the RG being at risk of learning difficulties. Therefore, healthcare professionals need to identify students at increased risk of digital addiction and supervise them more closely to prevent the development of learning disabilities.

There are some limitations to this study. First, we targeted students in one country, so the results may not be generalizable to other regions and countries. For generalizing the results to other countries, the results must be confirmed through repeated studies. In addition, brainwaves are constantly changing, so it is necessary to diversify measurement methods, such as increasing the measurement time or measuring participants several times. Education-related factors must also be considered, such as educational achievements and previous knowledge. Third, in this study, the usage time for digital devices for the WRG was greater than that for the RG. This is because learning time, as well as leisure activities, was considered to contribute to digital device usage. In further research, groups that spend a large amount of time using digital devices must be compared, such as those that use digital devices for leisure activities such as games and play with those that use them for learning. Finally, it was difficult to identify the determinants of brainwave variation because this study focused on the comparison of WRG and RG. In future research, identifying the determinants of brainwave variation may be helpful for adolescents’ learning.

The results of the present study raised the necessity for further research on the relationship between digital media addiction and digital learning. These results were in close agreement with the results of previous studies, which reported that excessive media use can reduce students’ academic performance via increased behavioral problems in school [39,40]. However, since the use of digital devices has both advantages and disadvantages, the results need to be carefully analyzed. A previous study showed that the use of digital technology is controversial depending on the purpose and methods of usage [37]. As school health providers’ roles and responsibilities include supporting students’ health and advocating for prevention strategies to avoid harmful environments [41], healthcare professionals should be able to assess risk early and to systematically manage students. This management requires the development of interventions, such as digital learning programs, to provide advice to students and guide them. For this, school health providers must improve their competency in the use of neurophysical techniques such as brain activity analyses to educate patients and students [42].

## 5. Conclusions

In this study, we compared the differences in the levels of attention and relaxation during digital learning according to the risk of digital media addiction. We identified students at risk of digital media addiction, as well as those not at risk, and compared their levels of attention and relaxation when they learned using digital devices. The results of this study suggest that digital devices alter adolescents’ brain activity, providing information for healthcare professionals on how to guide adolescents in using digital learning methods. Through the results of this study, we were able to ascertain the necessity of further research related to risk of digital media addiction and learning difficulties. Further research is necessary to clarify the relationship between digital media addiction and learning effects. Based on the results of our study, healthcare professionals can develop programs for the safe use of digital learning among students and verify the effects of these programs.

## Figures and Tables

**Table 1:** Participants’ general characteristics and homogeneity between without-risk and risk groups (*N* = 83).

Characteristics		WRG (*n* = 54) *n* (%)	RG (*n* = 29) *n* (%)	χ^2^/t	*p*-Value
Gender	Male	24 (28.9)	19 (22.9)	3.356	0.067
	Female	30 (36.1)	10 (12.0)		
Age	Mean ± SD	14.67 ± 0.78	14.72 ± 0.80	−0.318	0.751
Experience of digital learning	Yes	24 (28.9)	13 (15.7)	0.001	0.973
	No	30 (36.1)	16 (19.3)		
Digital learning time (h/week)	Mean ± SD	3.40 ± 4.74	2.53 ± 4.03	0.831	0.408
Digital device usage time for rest (h/day)	Mean ± SD	1.65 ± 1.64	2.01 ± 1.56	−0.996	0.322
Health status	Healthy	51 (61.4)	26 (31.3)	0.645	0.422
	Unhealthy	3 (3.6)	3 (3.6)		
Experience of fatigue or pain with digital device usage	Yes	29 (34.9)	17 (20.5)	0.185	0.667
	No	25 (30.1)	12 (14.5)		

**Table 2:** Differences in attention between the without-risk and risk groups (WRG and RG, respectively) (*N* = 83).

Location	Before Digital Learning				After Digital Learning			
	WRG (*n* = 54)	RG (*n* = 29)	Z	*p*-Value	WRG (*n* = 54)	RG (*n* = 29)	Z	*p*-Value
Fp1	0.17 ± 0.15	0.21 ± 0.22	−0.91	0.364	0.19 ± 0.18	0.20 ± 0.15	−1.26	0.207
Fp2	0.19 ± 0.24	0.21 ± 0.26	−0.33	0.738	0.20 ± 0.23	0.20 ± 0.19	−0.67	0.504
F3	0.40 ± 0.17	0.48 ± 0.27	−0.95	0.344	0.45 ± 0.22	0.49 ± 0.25	−0.74	0.462
F4	0.41 ± 0.19	0.49 ± 0.26	−0.98	0.325	0.47 ± 0.22	0.48 ± 0.27	0.13	0.894
T3	0.66 ± 0.34	0.83 ± 0.45	−1.83	0.067	0.73 ± 0.54	0.84 ± 0.41	−1.78	0.076
T4	0.85 ± 0.72	0.92 ± 0.71	−0.98	0.325	0.76 ± 0.48	0.84 ± 0.45	−0.97	0.330
P3	0.64 ± 0.28	0.69 ± 0.37	−0.45	0.654	0.78 ± 0.33	0.75 ± 0.34	0.38	0.702
P4	0.68 ± 0.29	0.75 ± 0.40	−0.57	0.567	0.79 ± 0.31	0.82 ± 0.43	−0.17	0.864

**Table 3:** The differences between before and after digital learning in the level of attention and relaxation between the risk and without-risk groups.

Location	Attention				Relaxation			
	WRG (*n* = 54)	RG (*n* = 29)	Z	*p*-Value	WRG (*n* = 54)	RG (*n* = 29)	Z	*p*-Value
Fp1	0.02 ± 0.10	−0.01 ± 0.17	−0.48	0.633	−0.06 ± 1.20	0.18 ± 2.25	−0.78	0.434
Fp2	0.00 ± 0.10	−0.00 ± 0.20	−1.36	0.175	−0.24 ± 1.35	0.24 ± 3.04	−0.19	0.626
F3	0.05 ± 0.13	0.02 ± 0.21	0.17	0.640	−0.06 ± 1.67	0.60 ± 2.42	−2.16	0.031
F4	0.06 ± 0.12	−0.00 ± 0.22	1.16	0.248	−0.28 ± 1.50	0.70 ± 2.34	−2.39	0.017
T3	0.07 ± 0.36	0.01 ± 0.39	0.18	0.856	0.24 ± 1.32	0.33 ± 1.69	−0.09	0.932
T4	−0.08 ± 0.59	−0.08 ± 0.48	0.55	0.580	0.24 ± 1.30	0.58 ± 1.73	−0.97	0.335
P3	0.14 ± 0.23	0.57 ± 0.22	0.75	0.456	−0.20 ± 2.77	0.25 ± 3.90	−1.08	0.281
P4	0.11 ± 0.24	0.07 ± 0.27	0.46	0.647	−0.35 ± 2.30	0.52 ± 4.45	−1.38	0.169

## Data Availability

The data that support the findings of this study are available from the corresponding author upon reasonable request.
